# Interference in transcription of overexpressed genes by promoter-proximal downstream sequences

**DOI:** 10.1038/srep30735

**Published:** 2016-08-03

**Authors:** A. Turchinovich, H. M. Surowy, A. G. Tonevitsky, B. Burwinkel

**Affiliations:** 1 Molecular Epidemiology C080, German Cancer Research Center, Im Neuenheimer Feld 581, Heidelberg, Germany; 2 Molecular Biology of Breast Cancer, Department of Gynecology and Obstetrics, Im Neuenheimer Feld 440, Heidelberg, Germany; 3 P. Hertsen Moscow Oncology Research Institute, National Center of Medical Radiological Research, 3 Second Botkinsky Lane, Moscow, 125284, Russia

## Abstract

Despite a high sequence homology among four human RNAi-effectors Argonaute proteins and their coding sequences, the efficiency of ectopic overexpression of AGO3 and AGO4 coding sequences in human cells is greatly reduced as compared to AGO1 and AGO2. While investigating this phenomenon, we documented the existence of previously uncharacterized mechanism of gene expression regulation, which is manifested in greatly varying basal transcription levels from the RNApolII promoters depending on the promoter-proximal downstream sequences. Specifically, we show that distinct overexpression of Argonaute coding sequences cannot be explained by mRNA degradation in the cytoplasm or nucleus, and exhibits on transcriptional level. Furthermore, the first 1000–2000 nt located immediately downstream the promoter had the most critical influence on ectopic gene overexpression. The transcription inhibiting effect, associated with those downstream sequences, subsided with increasing distance to the promoter and positively correlated with promoter strength. We hypothesize that the same mechanism, which we named promoter proximal inhibition (PPI), could generally contribute to basal transcription levels of genes, and could be mainly responsible for the essence of difficult-to-express recombinant proteins. Finally, our data reveal that expression of recombinant proteins in human cells can be greatly enhanced by using more permissive promoter adjacent downstream sequences.

Small non-coding regulatory RNAs including siRNAs and miRNAs mediate their function in association with proteins of Argonaute (AGO) family^1^,^2^. Besides their well described function in regulating targeted mRNAs stability and/or translation, Argonaute proteins also protect miRNAs from degradation in nuclease-rich environments including extracellular body fluids^3^,^4^,^5^. In addition, there are strong indications that Argonaute-miRNA complexes regulate gene expression in the nucleus of human cells and could be responsible for establishing targeted epigenetic chromatin modifications^6^,^7^,^8^. Human cells express four Argonaute genes (AGO1, AGO2, AGO3, and AGO4) of well conserved length and exon-intron structure. They share 77–84% amino acid sequence identity with each other and 70–76% nt identity of the CDSs^2^. Pioneering mRNA/miRNA pull-down experiments showed that similar transcripts are bound to different AGO proteins and suggested that four human Argonautes could be functionally redundant^9^,^10^. However, AGO2 is the only Argonaute capable of cleaving targeted mRNA transcripts^9^,^11^ and is also indispensable for biogenesis of some miRNAs^12^. Functional redundancy of at least AGO1, AGO2 and AGO3 proteins has been further questioned by a number of studies demonstrating that many miRNAs have strong biases towards the association with a particular Argonaute in human cells^5^,^13^,^14^. Significant tissue-specific differences in relative expression of Argonautes genes and robust changes of their expression during mammalian development also speak against functional redundancy of Argonaute proteins^15^,^16^,^17^.

In mammalian tissues Argonaute genes are expressed in different proportions depending on cell type and differentiation stage^2^,^15^,^16^,^18^, however in most human cell types AGO3 and AGO4 are expressed at much lower levels than AGO1 and AGO2^18^,^19^. Interestingly, the efficiency of exogenous overexpression of AGO3 and AGO4 coding sequences in cultured cells was also found to be dramatically lower as compared to AGO1 and AGO2 both on protein and mRNA levels^9^,^18^,^20^,^21^. These observations have generated a hypothesis that certain elements within AGO coding sequences may impair their expression efficacy *in vivo*. Thus, some authors suggested that differential distribution of rare codons over AGO3 and AGO4 coding sequences may enhance hydrolysis of their mRNAs in the cytoplasm during translation^18^,^21^. Indeed, this hypothesis would also explain lower expression of endogenous human AGO3 and AGO4 mRNAs as compared to AGO1 and AGO2^18^.

In this work, we confirm that AGO3 and AGO4 coding sequences are overexpressed with considerably lower efficacy as compared to AGO1 and AGO2 on both mRNA and protein levels in human cells. Furthermore, we demonstrate that impaired overexpression efficiency of AGO3 and AGO4 protein coding transcripts is independent on their translation on ribosomes and is manifested due to distinct velocity of mRNA synthesis in the cell nucleus. We also confirm that sequence-specific targeting via endogenous RNAi pathways cannot be responsible for this phenomenon. Interestingly, different regions of human AGO1, AGO2, AGO3 and AGO4 coding sequences had strong and varied impact on the amount of transcripts generated from CMV promoter when subcloned immediate downstream the promoter. Strikingly, the transcription efficacy among even highly homologous sequences varied up to 10–50 folds. At the same time, the observed impacts of conventional regulatory DNA sequences, including upstream enhancer of CMV promoter, Sp1 and Ap1 binding regions, on the transcription rate were much more modest. Finally, the inhibitory effects of downstream sequences were strongly dependent on their distance to the promoter and positively correlated with promoter strength. Based on this data, we suggest a hypothesis that retardation of RNApolII elongation on certain promoter-proximal DNA sequences could cause its collision with the following polymerases, thus prematurely terminating transcription. While in more distant regions from the promoter the processing rate of RNA polymerase could become more even or robust, this effect is only evident at the promoter proximal downstream sequences. It is feasible that besides the strength of a promoter, promoter-proximal downstream sequences could significantly contribute to basal transcription rate of many genes.

## Results

### Impaired expression of ectopic AGO3 and AGO4 mRNAs and proteins in human cells

**Figure 1:**
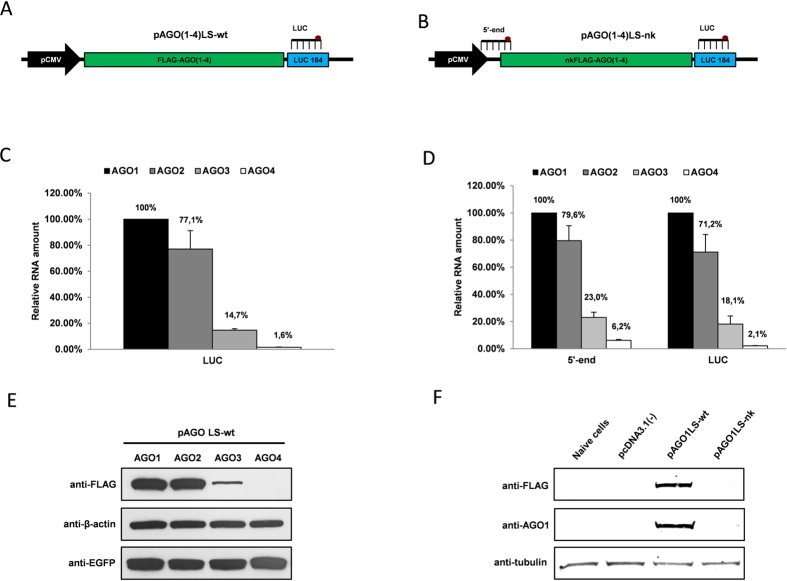
Different overexpression efficiency of human Argonaute coding sequences. (**A**) Structure of pAGO1LS-wt, pAGO2LS-wt, pAGO3LS-wt and pAGO4LS-wt used for transient overexpression of recombinant human Argonautes coding regions containing N-terminal FLAG-tag. The location of the luciferase TaqMan qPCR probe (LUC) is indicated on the plasmid map. (**B**) Structure of pAGO1LS-nk, pAGO2LS-nk, pAGO3LS-nk and pAGO4LS-nk encoding protein synthesis-impaired AGO cDNAs (lacking Kozak sequences and the first 2 nucleotides in the starting ATG codons). The location of the 5′-end and 3′-end (LUC) TaqMan qPCR probes is indicated on the plasmid map. TaqMan qRT-PCR analysis was used to estimate the relative overexpression of recombinant Argonaute transcripts generated from pAGO(1–4)LS-wt (**C**) and pAGO(1–4)LS-nk plasmids (**D**) in U2OS cells 24 h after transient transfection. The qPCR data is presented as mRNA levels relative to cells transfected with AGO1 encoding plasmids, and normalized on the NeoR mRNA levels in each sample. Each bar represents mean + S.D of three independent transfections. **(E**) Western immunoblotting demonstrating expression of FLAG-AGO1, FLAG-AGO2, FLAG-AGO3, FLAG-AGO4, EGFP and β-actin proteins in U2OS cells 24 h after transfection with pAGO1LS-wt, pAGO2LS-wt, pAGO3LS-wt and pAGO4LS-wt vectors mixed with equal amount of pEFGP-C1 plasmid. (**F)** Western immunoblotting performed with anti-FLAG and anti-AGO1 antibodies demonstrating the absence of recombinant AGO1 protein production in U2OS cells after transfection with pAGO1LS-nk plasmid, in contrast to cells transfected with pAGO1LS-wt.

We initiated this study after making an independent observation of the markedly different overexpression efficiency of human Argonautes coding sequences from an ectopic CMV promoter, both on mRNA and protein level in several common human cell lines (Fig. 1A,C,E; Supplementary Material 1)^9^,^18^. Despite the fact that all four AGO coding sequences share a high degree of homology, the overexpression of AGO1 and AGO2 was significantly stronger as compared to AGO3 and AGO4. Specifically, when the overexpression of FLAG-AGO mRNAs was normalized on the expression of NeoR gene located under a separate SV40 promoter in the same constructs relative AGO1: AGO2: AGO3: AGO4 mRNA abundances constituted 100%: 77.1%: 14.7%: 1.6%. The level of AGO3 protein was impaired proportionally to its mRNA level as compared to AGO1 and AGO2, while AGO4 expression was below the detection limit on western blot (Fig. 1E). Interestingly, the levels of endogenous AGO3 and AGO4 transcripts in U2OS, and other tested cell lines, were markedly reduced compared to AGO1 and AGO2 (Supplementary Material 2)^18^. These observations suggested that the reduced expression of AGO3 and AGO4 genes could be due to impaired mRNA stability in the cytoplasm, or sequence-specific targeting of their coding sequences. Since all Argonautes-coding sequences were cloned into the same expression vector under the same promoter, a contribution of a differential splicing and/or regions of Argonaute genes outside coding sequences themselves (including promoters, introns, CpG islands, 5′-UTR, 3′-UTR, transcription factor binding sites, silencers and enhancers) to this phenomenon can be excluded.

Previously, Grimm and colleagues hypothesized that a suboptimal rare codons distribution throughout the AGO3 and AGO4 mRNAs could explain the reduced translation efficiency of these proteins^21^. While Valdmanis and co-authors also found exogenous AGO3 and AGO4 mRNA levels to be considerably reduced^18^, a suboptimal codons structure could still influence mRNA stability by inducing ribosome pausing and thus increasing the accessibility of RNA-instability elements^22^,^23^. To test whether translation on ribosomes could contribute to differential expression of Agronaute mRNAs, we generated expression vectors carrying FLAG-AGO coding inserts without Kozak motifs and the first two nucleotides in the initiating codons and, therefore, unable to initiate translation (Fig. 1B,F). The resulting plasmids, designated as pAGO1LS-nk, pAGO2LS-nk, pAGO3LS-nk and pAGO4LS-nk, were transiently overexpressed in U2OS cells, and relative AGOs mRNAs levels were normalized on NeoR mRNA expression after qRT-PCR. As in the case with wild-type Argonautes (Fig. 1A), the overexpression of protein-null AGO3 and AGO4 mRNAs were impaired to similar levels as in wild-type constructs (Fig. 1B,D; Supplementary Material 1). These results strongly suggest the reduced levels of both ectopically overexpressed and endogenous AGO3 and AGO4 cannot be fully attributed to varying translation efficiencies on ribosomes (Supplementary Material 2).

### Nuclear pathways are responsible for reduced ectopic overexpression of AGO3 and AGO4 mRNAs in human cells

**Figure 2:**
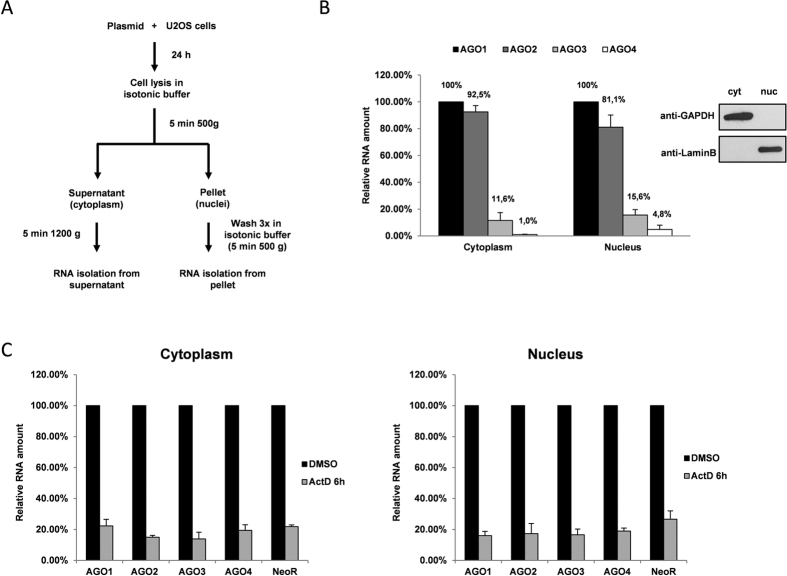
Nuclear pathways are responsible for the reduced ectopic overexpression of AGO3 and AGO4 mRNAs in human cells. (**A**) Schematic representation of the experimental procedure of U2OS cells fractionation into nuclear and cytoplasmic fractions after transfection with recombinant constructs. (**B**) (*left*) TaqMan qRT-PCR analysis demonstrating relative overexpression of recombinant Argonaute transcripts in cytoplasmic and nuclear fractions after transfection of U2OS cells with pAGO(1–4)LS-nk constructs. The qPCR data is presented as mRNA levels relative to cells transfected with AGO1 encoding plasmids, and normalized on the NeoR mRNA levels in each sample. (*right*) Western immunoblotting demonstrating the absence of cytoplasmic GAPDH protein in nuclear cell fractions and nuclear LaminB protein in cytoplasmic fractions. (**C**) The amounts of AGO1, AGO2, AGO3, AGO4 and NeoR recombinant transcripts in the cytoplasm (left) and nuclear (right) fractions in pAGO(1–4)LS-nk transfected U2OS cells after treatment with actinomycin D for 6 hours (relative to cells treated with DMSO). The qRT-PCR data is presented as mRNA levels for each transcript type relative to cells treated with DMSO, and normalized on the levels of spiked-in synthetic cel-miR-39 control. Note, the half-lives of all transcripts are similar in both cytoplasm and the nuclei.

We next tested whether nuclear mechanisms could contribute to uneven expression of ectopic AGO mRNAs. For this purpose, nuclear and cytoplasmic RNA fractions were isolated from U2OS cells 24 hours post-transfection with pAGO(1–4)LS-nk plasmids (Fig. 2A). Subsequent qRT-PCR analysis revealed that the relative abundance of AGO3 and AGO4 transcripts were proportionally lower as compared to AGO1 and AGO2 both in cytoplasmic and nuclear fractions (Fig. 2B). This indicated that either decreased mRNA synthesis or/and faster mRNA degradation in the nucleus could be mainly responsible for lower amounts of ectopic AGO3 and AGO4 mRNA. When transcription was blocked by incubating the transfected U2OS cells with 10 μg/mL actinomycin D for 6 hours, all four overexpressed AGO genes demonstrated a similar reduction in mRNA levels over time both in the cytoplasm and the nucleus (Fig. 2C). This suggests that mRNA synthesis rate rather than mRNA hydrolysis is the main factor in the reduced ectopic overexpression of AGO3 and AGO4 cDNAs (Fig. 2C).

### Known nuclear mechanisms of gene expression regulation cannot explain impaired ectopic expression of AGO3 and AGO4 coding regions

Alternative polyadenylation is one of the well-known mechanisms of differential expression of pre-mRNAs in the nucleus. However, it could not explain the observed differences in exogenous AGO expression, because relative abundance of 5′-end promoter-proximal transcripts were similar to the abundance of 3′-end transcripts (Fig. 1D; Supplementary Material 1). Premature transcription termination on Argonatues coding sequences is another putative mechanism which cannot be excluded, provided that the terminated transcripts are rapidly degraded. Recently, it has been shown that the microprocessor complex consisting of Drosha/DGCR8 orchestrates the recruitment of several termination factors to initiate RNAPII pausing and premature termination at the HIV-1 promoter through cleavage of the stem-loop RNA TAR^24^. However, dual siRNA silencing of microprocessor components Drosha and DGCR8 did not affect the pattern of AGO1-4 overexpression (Supplementary Material 3). We also excluded hyper-methylation of the ectopic vectors as a cause for the reduced AGO3 and AGO4 overexpression efficiency since their relative abundance did not change after de-methylating agent 5-aza-2′-deoxycytidine was added during transfection (Supplementary Material 4).

### The first 1000 nt downstream the promoter significantly affect gene expression rate

**Figure 3:**
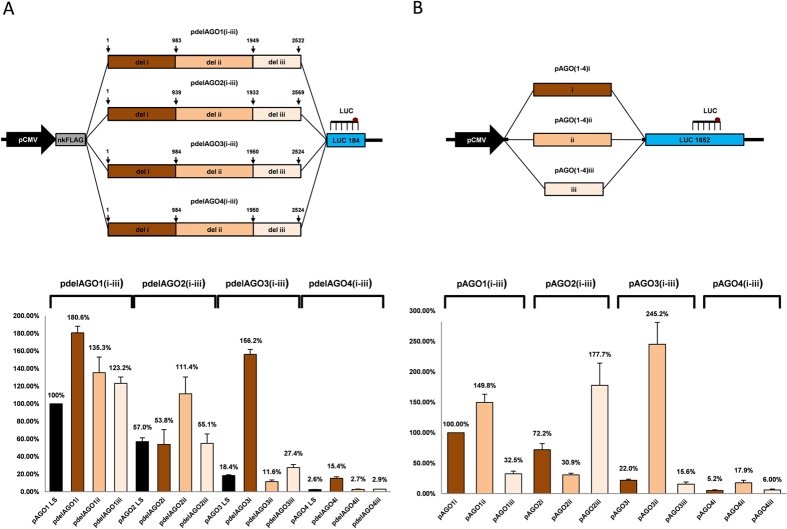
The first 1000 nt downstream the promoter significantly affect gene expression rate. (**A**) (*Upper part*): Structure of pdelAGO(1–4)i, pdelAGO(1–4)ii and pdelAGO(1–4)iii, the deletion mutants of the pAGO(1–4)LS-nk plasmids, used for transient overexpression of deleted AGO coding sequences. The location of each deleted region (i, ii or iii) is shown on plasmid DNA map for each AGO gene. (*Lower part*): TaqMan qPCR analysis of relative overexpression of exogenous transcripts 24 h after transfection of parental pAGO(1–4)LS-nk and deleted constructs in U2OS cells. (**B**) (*Upper part):* Structure of pAGO(1–4)i, pAGO(1–4)ii and pAGO4(1–4)iii used for transient overexpression of sequential parts of AGO coding regions upstream the full-length luciferase gene. The overexpressed AGO regions (i, ii, iii) corresponded exactly to the deleted regions in the pdelAGO(1–4) constructs. (*Lower part):* TaqMan qPCR analysis of relative overexpression of exogenous transcripts 24 h after transfection of U2OS cells with pAGO(1–4)i, pAGO(1–4)ii and pAGO(1–4)iii. All qPCR data is presented as mRNA levels relative to cells transfected with full-length AGO1 encoding plasmids (**A**) or region i of AGO1 (**B**), and normalized on the NeoR mRNA levels for each transfection. Each bar represents mean + S.D of three independent transfections.

Next, we aimed to determine which parts of the Argonautes coding sequences of approximately 2.5 kb total length could be responsible for their differential ectopic transcription. For this purpose we generated FLAG-AGO overexpression plasmids containing one of the three sequential deletions within their corresponding AGO encoding sequences (Fig. 3A). Expression of deleted and full-length AGO sequences were analyzed with a TaqMan assay targeting the downstream luciferase gene fragment (Fig. 3A). Deletion of any of the three parts of AGO1 and AGO2 did not negatively affect their transcription rate, indicating that higher overexpression efficiency of AGO1 and AGO2 relative to AGO3 and AGO4 was not due to enhancing effects of their coding sequences on transcription rate (Fig. 3A). Interestingly, deleting either middle or C-terminal parts of AGO coding sequences did not considerably alter their relative overexpression pattern, indicating that the very promoter-proximal regions were mainly responsible for the impaired AGO3 and AGO4 transcription (Fig. 3A). In contrast, a dramatic 8.5-fold increase of AGO3 transcription was observed when the promoter-proximal part (approximately 1000 nt) was deleted from the AGO3 coding sequence (pdelAGO3i), restoring the ectopic expression of AGO3 even above the levels of the full-length AGO1. Similarly, deletion of the first 1000 nt region from the AGO4 cDNA elevated the amount of the transcript approximately 6-fold (pdelAGO4i) compared to full-length AGO4, however, final expression of deleted AGO4 transcript was still restored to only 15.4% as compared to full-length AGO1.

The fact that the restoration of AGO4 mRNA was weaker after deleting the promoter-proximal 1 kb region of AGO4 cDNA indicates that the adjacent middle portion of the AGO4 coding sequence could also be inhibitory for transcription when located immediately downstream the promoter. To test this idea we cloned the portions of AGO coding sequences corresponding to the deleted AGO regions directly under the CMV promoter upstream the full-length luciferase gene (pAGO(1–4)(i-iii), Fig. 3B). Indeed, the middle region of the AGO4 coding sequence also significantly inhibited the downstream luciferase expression (as compared to the N-terminal region of AGO1), however the inhibition was approximately 3-fold weaker than that of the AGO4 N-terminal region (Fig. 3B). In strong contrast to this, the equivalent middle region of AGO3 had approximately 10-fold higher transcription efficacy as compared to the N-terminal region of AGO3. Thus, both the deletion mutagenesis (Fig. 3A) and the AGO CDS parts overexpression data (Fig. 3B) consistently demonstrate that a promoter-adjacent sequence has a determining effect on transcription rate of the whole mRNA. Interestingly, the 12 different fragments from the four AGO coding sequences showed strong and unexpectedly varied effects on the downstream luciferase expression (Fig. 3B). Even highly homologous sequences (e.g. regions AGO1i and AGO4i; regions AGO2iii and AGO4iii; regions AGO3ii and AGO4ii having sequence identities 73%, 73% and 77% respectively) showed 15–50 fold variation in transcription rates when cloned downstream the ectopic CMV promoter.

We further found that the overexpression efficiency of recombinant full-length AGO transcripts varied 2-fold depending on whether a short 200 bp EGFP gene fragment was cloned in either forward or reverse orientation immediately downstream the CMV promoter (Supplementary Material 5). This underlines that the observed promoter-proximal inhibition effect is not a distinguished feature of the four Argonautes coding sequences, and is also independent of a transcript length and the overall GC composition of a downstream sequence.

### The distance of AGO3 and AGO4 coding sequences to the CMV promoter is critical for their expression in human cells

**Figure 4:**
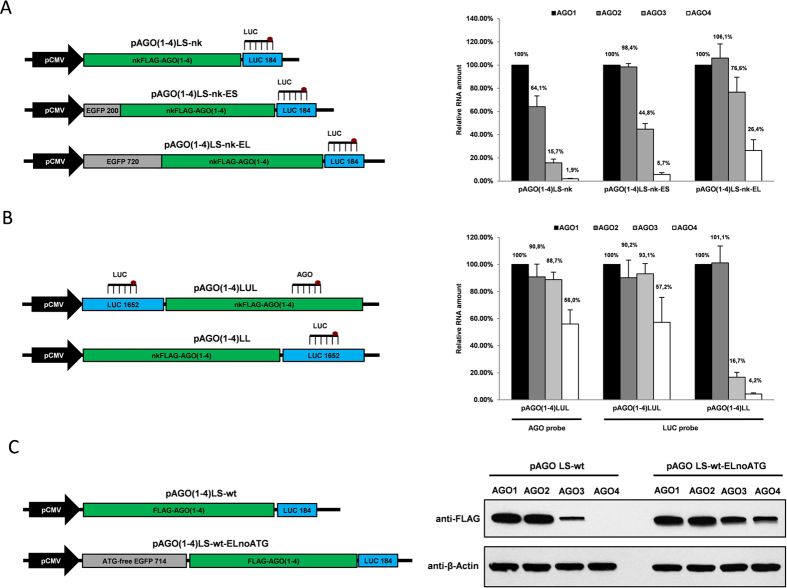
The distance of AGO3 and AGO4 coding sequences to the CMV promoter is critical for their expression in human cells. (**A**) (*Left*): Structure of pAGO(1–4)LS-nk, pAGO(1–4)LS-nk-ES and pAGO(1–4)LS-nk-EL used for transient overexpression of AGO coding regions containing either short 200 bp or full-length 720 bp EGFP gene (without the initiating ATG codon) respectively upstream. (*Right*): TaqMan qRT-PCR analysis of recombinant mRNAs generated from these plasmids in U2OS cells 24 h after transfection. (**B**) (*Left*): Structure of pAGO(1–4)LUL containing full-length 1652 bp luciferase gene (without the initiating ATG codon) upstream the AGO coding sequences, and pAGO(1–4)LL plasmids containing the same 1652 bp luciferase gene fragment downstream the AGO coding sequences. (*Right*): TaqMan qRT-PCR analysis of recombinant mRNAs generated from these plasmids in U2OS cells 24 h after transfection. The location of luciferase (LUC) and Argonaute-specific (AGO) TaqMan qPCR assays used is indicated on the corresponding plasmid map. All qPCR data is presented as mRNA levels relative to cells transfected with AGO1 encoding plasmids, and normalized on the NeoR mRNA levels for each transfection. Each bar represents mean + S.D of three independent transfections. (**C**) (*Left*): Structure of the parental pAGO(1–4)LS-wt and the derivative pAGO(1–4)LS-wt-ELnoATG construct containing the 714 bp EGFP cDNA deprived from all ATG codons upstream the AGO coding regions. (*Right*): Western immunoblot performed with anti-FLAG and anti-β-actin antibody showing FLAG-AGO1, FLAG-AGO2, FLAG-AGO3, FLAG-AGO4 and actin proteins expression in U2OS cells 24 h after transfection with pAGO(1–4)LS-wt and pAGO(1–4)LS-wt-ELnoATG.

To further corroborate that promoter-adjacent downstream sequences can critically affect transcription rate, we investigated whether the inhibitory effects of AGO3 and AGO4 sequences can be exempted by increasing their distance to the promoter. In accordance with this hypothesis, cloning of either truncated EGFP CDS (200 bp, region containing TaqMan assay probes and primers binding site) or protein-null full-length EGFP CDS (720 bp) upstream the FLAG-AGO encoding sequences restored the ectopic expression of both AGO3 and AGO4 approximately proportionally to the length of the upstream insert (Fig. 4A). Specifically, when the 720 bp EGFP insert was sub-cloned between the CMV promoter and the AGO sequences, the overexpression efficacy of AGO4 transcript constituted already 26.4% as compared to AGO1, while in parental pAGO(1–4)LS-nt vectors this ratio was 1.9% (Fig. 4A). Furthermore, AGO1, AGO2 and AGO3 overexpression efficacies became similar (Fig. 4A). Finally, inserting the full-length 1652 bp protein-null luciferase CDS fragment upstream the AGO coding sequences restored AGO3 overexpression to the levels of AGO1 and AGO2, while relative amount of AGO4 transcript constituted 57% of that of AGO1 (Fig. 4B). In contrast, full-length luciferase insertion downstream the AGO coding sequences yielded overexpression efficacy ratios similar to the parental vectors (Figs 1D and 4A,B).

These observations were also confirmed on the protein level. The overexpression capacities of recombinant FLAG-AGO3 and FLAG-AGO4 proteins were also restored to the levels of FLAG-AGO1 and FLAG-AGO2 after the full-length 714 bp EGFP gene fragment devoid of all internal ATG codons was inserted into pAGO(1–4)-wt plasmids directly downstream the promoters (Fig. 4C). Importantly, the upstream wild-type EGFP coding sequence (containing six internal ATG codons) mediated significantly less robust overexpression of Argonaute proteins, presumably due to reduced frequency of translation initiation on the FLAG-AGO initiating codons (Supplementary Material 6). The observation that the incidence of ATG codons upstream the ORF can significantly affect translation also fits into the currently accepted model of eukaryotic translation initiation^25^.

The fact that the expression of recombinant FLAG-AGO3 and FLAG-AGO4 sequences increased with their distancing from the ectopic CMV promoter further confirms the ability of promoter-adjacent downstream DNA to significantly affect the expression of the whole transcript. Notably, the currently well-characterized mechanisms of gene expression regulation in the nucleus including alternative polyadenylation, alternative splicing and RNA interference would not explain the observed phenomena, since none of those mechanisms relies significantly on the proximity to the promoter.

### The impact of promoter proximal downstream sequences on transcription is strongly dependent on promoter strength

**Figure 5:**
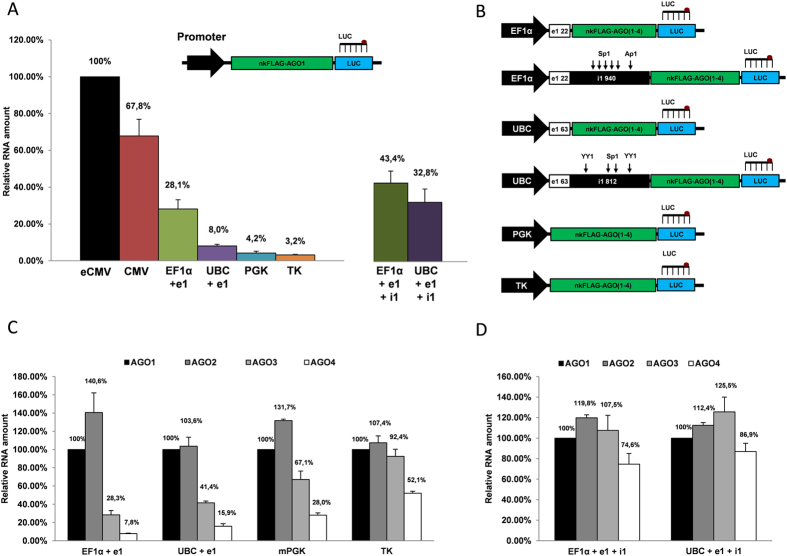
The impact of promoter proximal downstream sequences on transcription is strongly dependent on promoter strength. (**A**) Structure of recombinant construct used for overexpression of FLAG-AGO1 transcript under eCMV, CMV, EF1α + e1, UBC + e1, PGK, TK, EF1α + e1 + i1 and UBC + e1 + i1 promoters and qRT-PCR analysis of recombinant FLAG-AGO1 transcripts generated from those plasmids in U2OS cells 24 hour after transfection. Data is presented as a graph combining relative overexpression efficacy of transcripts from plasmids with eCMV promoter (taken as 100%) vs. transcripts overexpressed from other promoters. Each bar represents mean + S.D of three independent replicates. (**B**) Structure of recombinant constructs used for overexpression of FLAG-AGO1, FLAG-AGO2, FLAG-AGO3 and FLAG-AGO4 transcripts under EF1α + e1, UBC + e1, EF1α + e1 + i1, UBC + e1 + i1, PGK and TK promoters with indicated sizes of exon1 (e1) and intron1 (i1) and a number of Sp1, Ap1 and YY1 binding motifs within the introns. (**C**) TaqMan qPCR analysis of recombinant mRNAs generated from intron-free EF1α + e1, UBC + e1, PGK and TK promoters in U2OS cells 24 h after transfection. (**D**) TaqMan qPCR analysis of recombinant mRNAs generated from intron1 containing promoters EF1α + e1 + i1, UBC + e1 + i1 in U2OS cells 24 h after transfection. All qPCR data is presented as mRNA levels relative to cells transfected with AGO1 encoding plasmids, and normalized on the NeoR mRNA levels for each transfection. Each bar represents mean + S.D of three independent transfections.

To confirm that the inhibitory effect of AGO3 and AGO4 sequences was not restricted to only cytomegalovirus promoter, we have overexpressed human FLAG-AGO sequences from several other constitutive RNApolII promoters, including human elongation factor-1 alpha (EF1α), human ubiquitin C (UBC), mouse phosphoglycerate kinase 1 (mPGK) and herpex simplex virus thymidine kinase (TK) promoters, which are the most commonly used promoters for gene expression in mammals. We first compared the basal strengths of those promoters by measuring overexpression efficiency of AGO1 coding sequence in U2OS cells (Fig. 5A). The EF1α promoter was cloned into pAGO(1–4)LS-nk vectors directly upstream the AGO sequences (instead of eCMV promoter) either together with the first 22 bp exon (EF1α + e1) or with the first exon and the first 940 bp intron of human EF1α gene (EF1α + e1 + i1). Likewise, the UBC promoter was cloned either together with only the first 63 bp exon (UBC + e1) or with the first exon and the first 812 bp intron of human UBC gene (UBC + e1 + i1) (Fig. 5B). Subsequently, all plasmids were transfected into U2OS cells in culture, RNA was isolated 24 hour post-transfection and the relative expression of transcripts was analyzed with luciferase TaqMan qPCR Assay (Fig. 5A). As expected, the strongest expression of the FLAG-AGO1 transcript in U2OS cells was observed under the enhanced CMV (eCMV) promoter. Interestingly, deleting the upstream enhancer decreased the strength of the eCMV promoter only about 30%, what was much less pronounced as compared to the impacts of promoter-proximal downstream AGO sequences (Fig. 3B). Furthermore, the expression of FLAG-AGO1 mRNA under EF1α + e1 promoter corresponded to 28,1% of its expression from the eCMV promoter, while in the presence of intron (EF1α + e1 + 1) this value constituted 43,4%. Human UBC promoter (UBC + e1) demonstrated much weaker expression of the recombinant transcript (8% as compared to eCMV); however, the presence of the first 812 bp intron of human UBC gene (UBC + e1 + i1) mediated significantly higher expression (32,8% as compared to eCMV). The strength of the mPGM and TK promoters were notably lower and constituted only 4,2% and 3,2% as compared to eCMV respectively (Fig. 5A).

After transient overexpression of all protein-null FLAG-AGO sequences under EF1α + e1, EF1α + e1 + i1, UBC + e1, UBC + e1 + i1, PGM or TK promoters in U2OS cells, the relative levels of each Argonaute transcript was analyzed with luciferase TaqMan assay (Fig. 5B–D). Surprisingly, differences in relative Argonautes overexpression efficacies diminished proportionally with the decreasing promoters strengths (Fig. 5A,C). Thus, under the weakest TK promoter, the relative expression of FLAG-AGO1, FLAG-AGO2 and FLAG-AGO3 transcripts were similar, while the expression of FLAG-AGO4 was impaired to only 50% of that of FLAG-AGO1. Furthermore, Argonautes overexpression efficacy from introns containing EF1α and UBC promoters was approximately equal (Fig. 5D). This data strongly suggests that (1) interference in genes overexpression by downstream promoter proximal sequences is not restricted to the CMV promoter, and could be a universal phenomenon, and (2) sequences immediately downstream the RNApolII promoters including endogenous introns might have a determining effect on transcription rates of mRNAs. The fact that certain promoter-proximal introns may enhance mRNA accumulation has been known before, however, the mechanisms behind this phenomenon was thought to be mRNA stabilization and increased export into the cytoplasm due to splicing events^26^.

Our data also suggests that common downstream transcription factors binding sites including Sp1 and Ap1 had rather marginal effect on transcription from EF1α promoter. The presence of endogenous EF1α intron1 carrying five Sp1 and one Ap1 binding site increased the recombinant FLAG-AGO1 expression from EF1α promoter only 1.5-fold (Fig. 5A,B). The transcription of FLAG-AGO1 from the UBC + e1 + i1 promoter, which contained a downstream intron with two Sp1 and two YY1 transcription factors binding sites, was about 4 times stronger as compared to UBC + e1 promoter lacking the intron (Fig. 5A,B). However, the increases of transcription efficiencies from both EF1α + e1 + i1 and UBC + e1 + i1 promoters were significantly more profound for FLAG-AGO3 and FLAG-AGO4 transcripts, most likely due to a negation of the inhibitory influence of AGO3 and AGO4 sequences by their downstream distancing (see Figs 4 and 5C,D).

**Figure 6:**
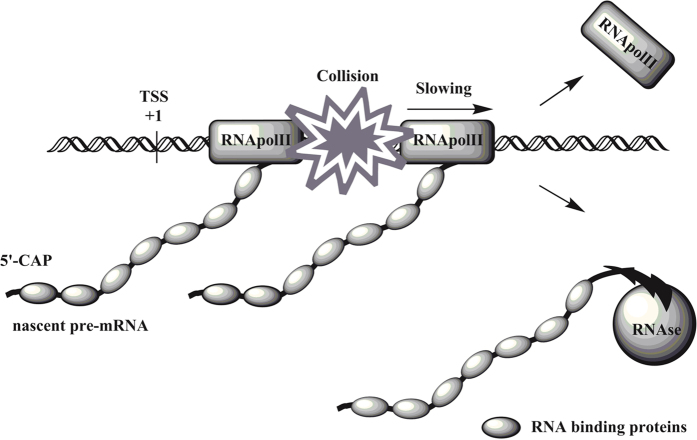
The putative mechanism of promoter proximal inhibition. Hypothetical mechanism demonstrating how promoter-proximal DNA could negatively affect the transcription of mRNA. Due to promoter proximal downstream RNApolII pausing, the following RNApolII collides with the slowed-down leading RNApolII what leads to dissociation of transcriptional machinery from DNA, and rapid degradation of nascent mRNA transcripts by RNAses.

Despite a significant (70–76% on DNA level) homology between four human full-length Argonaute CDSs (Supplementary Material 7A) the remaining differences in their sequences are sufficient to mediate the observed variation in overexpression capacities. For instance, AGO3ii and AGO4ii regions demonstrated approximately 14-fold difference in overexpression efficiency while having 77.1% sequence identity (Supplementary Material 7B). By analyzing particularities within Argonautes sequences and their parts we could not dissect any specific motifs or other features including prominent differences in nucleotides distribution (G/C and A/T content), which would correlate with lower or higher expression efficiency of certain promoter-proximal downstream sequences. The well-described promoter-downstream regulatory elements which are known to affect transcription rates include Sp1, Ap1 and YY1 binding sites, however, none of the four human AGO coding sequences contain any of those. Notably, the features within the AGO3 and AGO4 coding sequences show a considerably stronger effect on transcription rate than common regulatory sequences such as upstream CMV enhancer or immediate downstream Sp1, Ap1 and YY1 binding sites. Taking into account the fact that the repressing effect of downstream sequences subsides with decreasing promoter power (or frequency of transcription initiation), the most plausible hypotheses for this phenomenon are premature transcription termination or/and transcription pausing due to the collision of leading and following RNA polymerases within the first 1000–2000 nt downstream the promoter (Fig. 6). Indeed, the rate of RNApolII elongation differs depending on primary DNA sequence, and can be significantly slower at the promoter-proximal downstream regions^27^,^28^. According to this hypothesis, certain promoter-proximal sequences could facilitate RNApolII pausing and consequent collision of leading and following RNApolII complexes which results in premature transcription termination.

## Discussion

This work is primarily aimed to point out a surprising phenomenon which we encountered while investigating the putative mechanisms of differential overexpression capacity of Argonautes coding sequences in transfected human cells. Specifically, we demonstrate the existence of a hitherto uncharacterized mechanism of gene expression regulation which manifests in strong interference of promoter-proximal downstream DNA sequences in basal transcription rate from several constitutive RNApolII promoters. We found that different regions of human AGO1, AGO2, AGO3 and AGO4 coding sequences had strong and heterogenous impact on the amount of transcripts generated from CMV and other promoters when located downstream in close proximity to the promoter. Astoundingly, the difference in transcription efficacy among even highly homologous sequences was up to 10–50 folds. None of the currently known mechanisms could adequately explain this phenomenon, and we suggest a general hypothesis of how promoter proximal downstream DNA regions can affect transcription rate. Because an inhibitory effect of a given downstream sequence declined proportionally with the increasing distance to the promoter, this mechanism must harness the particularities of promoter adjacent sequences. Furthermore, inhibitory effects of downstream DNAs strongly correlated with the strength of the tested promoters, and only marginally manifested under very weak promoters. We hypothesize that certain downstream DNA sequences could facilitate RNA polymerase slowing or pausing, what in turn could lead to the increased incidence of collisions between leading and following RNA polymerases (Fig. 6). This theory would explain the immunity of very weak promoters to transcription inhibition by downstream DNA sequences, since lower frequency of transcription initiation would lead to a lower number of RNApolII molecules occupying a promoter-proximal DNA per fraction of time, therefore making collision events between polymerases less likely. Indeed several researchers has observed promoter proximal RNApolII pausing before, however, it remains unclear how broad this phenomenon occurs^27^. Many evidences indicate that RNApolII pausing during early elongation could be a widespread phenomenon in higher eukaryotes^28^,^29^, and that the processivity of elongating RNApolII is continually subject to regulation^30^,^31^. However, how exactly the efficiency of early elongation is regulated at the mechanistic and biochemical level remains unknown. Importantly, collisions of RNA polymerases can cause the enzymes and a nascent RNA to dissociate from the transcription machinery^32^,^33^. Finally, a number of recent studies have shown that some mammalian promoters can initiate transcription in both directions. It remains to be tested whether bidirectional transcription could contribute to the phenomenon of promoter proximal inhibition reported in this paper and affect the expression of protein-coding genes including human Argonautes^34^.

As a second aspect, our data suggest an efficient and easy approach to boost the production of difficult-to-express recombinant RNA and proteins. We show that certain protein coding DNA sequences can be suppressive for transcription especially from strong promoters and, as in the case of AGO4 (and to a lesser extent AGO3), the expression could be drastically increased by cloning a more permissive downstream DNA sequence between a promoter and a coding sequence. As an example we demonstrate that the full-length EGFP coding sequence devoid from ATG codons can serve as a highly permissive promoter-adjuscent sequence that considerably restores/improves both mRNA and protein expression rates.

Finally, our work introduces an update to the current knowledge on the regulation of endogenous human Argonautes genes. In almost all tissues and cell lines, the expression of AGO3 and AGO4 genes is lower as compared to AGO1 and AGO2. Initially, it was hypothesized that the same mechanism could be responsible for the impaired expression of both endogenous AGO3 and AGO4 mRNAs and ectopic AGO3 and AGO4 coding sequences^18^. Indeed, the existence of an RNA interference loop targeting coding regions within AGO3 and AGO4 genes would elegantly explain such a phenomenon. Likewise, a decreased stability of the AGO3 and AGO4 encoding mature mRNAs in the cytoplasm might have served as an alternative explanation. This opinion was supported by previous reports suggesting that differential distribution of rare codons may limit the accumulation of AGO3 and AGO4 mRNAs as compared to AGO1 and AGO2^18^. In these studies, a codon optimization strategy, which implied the introduction of several hundreds point mutations in the wild-type human AGO3 and AGO4 coding sequences, restored their mRNA and protein expression to the levels observed for wild-type AGO1 and AGO2^18^. The rare codons were found to be concentrated within the first half of the AGO1 and AGO2 coding sequences, while AGO3 and AGO4 mRNAs have an even distribution of rare codons along their open reading frames. The authors suggested a hypothesis that higher occurrence of rare codons in the beginning of the AGO1 and AGO2 transcripts may induce a more uniform positioning of ribosomes, thus facilitating ribosome movement and efficiency of translation^18^. In contrast, our data indicate that the impaired production of AGO3 and AGO4 mRNAs is mainly independent on their translation, what excludes the impact of rare codons distribution on regulation of their abundance. Furthermore, we demonstrate that inhibition of the exogenous AGO3 and AGO4 expression occurs mainly at the level of mRNA synthesis in the cell nucleus. However, our observations do not contradict the earlier studies since it is feasible that the significant changes introduced to Argonautes coding sequences during codon optimization^18^,^21^ amended the inhibitory influence of the promoter-proximal regions of wild-type AGO3 and AGO4 DNA on transcription. Unlike in constructs used for recombinant Argonautes overexpression in this study, in human genomic DNA Argonautes coding sequences are located in significantly larger distances from their endogenous promoters (Supplementary Material 8). Therefore, the mechanism of lower overexpression of ectopic AGO3 and AGO4 coding sequences is unlikely to be related to the mechanism sustaining the lower expression of endogenous AGO3 and AGO4 mRNAs. However, basal transcription rates of genes, including Argonautes, from endogenous promoters could be influenced by proximal downstream DNA in a similar manner. It remains to be tested to which extent the phenomenon of promoter proximal inhibition described in this work contributes to basal transcription level of genes in general; however it could have a significant practical impact on strategies to enhance overexpression efficiency of recombinant proteins.

## Materials and Methods

### Cell culture, transfection and fractionation

All cell lines were obtained from the American Type Culture Collection (ATCC). In addition, all cell lines have been genetically authenticated by DKFZ (Heidelberg) core facility. MCF7 cell line was grown in αMEM supplemented with 10% FBS, 1% non-essential amino acid and penicillin/streptomycin at 37 °C in 5% CO_2_. U2OS and HeLa cells were grown in DMEM supplemented with 10% FBS, 1% non-essential amino acid and penicillin/streptomycin at 37 °C in 5% CO_2_. For the overexpression of AGO constructs cells were transiently transfected with 300 ng of corresponding plasmid DNA using Turbofect transfection reagent (Fermentas) according to the manufacturer’s protocol. In some experiments 100 ng pEGFP-C1 vector was co-transfected with plasmids overexpressing wild-type Argonautes. Next day after transfection cells were lysed in either Laemmli loading buffer or Qiazol reagent and used for either Western blot analysis or total RNA isolation. Actinomycin D was purchased at Sigma-Aldrich (cat. A1410), dissolved in DMSO and added to cells at 10 μg/mL final concentration. Isolation of nuclear and cytoplasmic fraction was performed as described in^35^. Briefly, cells were washed with 1x PBS and lysed for 15 min at +4 °C in hypotonic buffer (10 mM Tris pH 7.5, 10 mM NaCl, 3 mM MgCl2, 0.3% NP-40 and 10% glycerol) containing RNAsin (Promega) and the cytoplasm fractions were collected. The remaining nuclei were centrifuged for 5 min at 500 g and washed 3 times in the hypotonic buffer. Cytoplasm fractions were centrifuged 5 min at 1200 g to remove possible contamination by nuclei and lysed in Trisol LS reagent (Sigma) at 1:3 proportion. The washed nuclear fractions were lysed in 700 μL Qiazol reagent from miRNAeasy mit (Qiagen).

### Plasmid DNAs and deletion mutagenesis

To allow accurate quantification of Argonautes overexpression FLAG-AGO coding sequences were amplified from pIRESneo-FLAG/HA-AGO1, pIRESneo-FLAG/HA-AGO2, pIRESneo-FLAG/HA-AGO3 and pIRESneo-FLAG/HA-AGO4 plasmids originally described in^9^ with corresponding primers (Supplementary Material P1), and subcloned into pcDNA3.1(-) vector using XbaI/EcoRI sites which carries the NeoR gene under a separate SV40 promoter. This allowed to adequately accounting for variations in transfection efficacy between samples. Additionally, 184 bp fragment of the firefly luciferase gene (containing the binding site for the TaqMan qPCR Assay probe and primers) was inserted after the stop codons of the AGO sequences using EcoRI/BamHI sites and enabled precise quantification of the four different FLAG-AGO transcripts with a single luciferase TaqMan qPCR assay (the resulting plasmids were named pAGO1LS-wt, pAGO2LS-wt, pAGO3LS-wt and pAGO4LS-wt). To generate protein-null mutants, FLAG-AGO coding sequences without Kozak sequences and the first bases of the initiating ATG codons were amplified with corresponding primers (Supplementary Material P1) from pIRESneo-FLAG/HA-AGO plasmids and sub-cloned into pAGO1LS-wt vector using XbaI/EcoRI sites instead of wild-type AGO1 sequence (the resulting plasmids were named pAGO1LS-nk, pAGO2LS-nk, pAGO3LS-nk and pAGO4LS-nk). To generate pAGO1LL, pAGO2LL, pAGO3LL and pAGO4LL vectors, 1652 nt full length luciferase gene (lacking initiating ATG codon) was amplified from pG5luc plasmid and cloned into pAGO1LS-nk, pAGO2LS-nk, pAGO3LS-nk and pAGO4LS-nk vectors instead of short 184 bp luciferase fragment downstream FLAG-AGO sequences via EcoRI/BamHI site. The constructs pAGO1LUL, pAGO2LUL, pAGO3LUL and pAGO4LUL were generated by cloning the full length 1652 bp luciferase sequence (lacking the initiating ATG codons) into pcDNA3.1(-) vector directly upstream the protein-null FLAG-AGO sequences via XbaI site. Cloning of either short (200 nt), full-length (720 nt) protein-null (without the first ATG codon and the ATG codon at the ORF in the middle) or full-length (714 nt) EGFP coding sequence completely devoid of ATG-codons was performed via XbaI site located before the FLAG-AGO coding sequences in the pAGO(1–4)LS-wt or pAGO(1–4)LS-nk after PCR amplification of the inserts from pEGFP-N plasmid and subsequent site-directed mutagenesis. Three different fragments of each four AGO sequences were cloned upstream the full-length luciferase sequence into the pAGO1LL plasmids instead of full-length AGO1 via XbaI/EcoRI sites after PCR amplification of inserts from original pIRESneo-FLAG/HA AGO(1–4) plasmids. Generation of pdelAGO1(i-iii)LS, pdelAGO2(i-iii)LS, pdelAGO3(i-iii)LS and pdelAGO4(i-iii)LS plasmids carrying deleted regions within AGO coding sequences were performed with Phusion Site-Directed Mutagenesis Kit (Thermo Scientific) according to the manufacturer’s protocol. All promoters were amplified from the corresponding plasmid DNAs (pEF-DEST51, pLVUT-tTR-KRAB, pLVPT-rtTR-KRAB-2SM2 and pRL-TK) and cloned into pAGO(1–4)LS-nk vectors instead of eCMV promoter via MluI/XbaI sites. The upstream CMV enhancer has been deleted from parental plasmid pAGO1LS-nk using Phusion Site-Directed Mutagenesis Kit (Thermo Scientific). All primers used in this work are listed in the Supplementary Material P1.

### Quantitative real-time PCR

Total RNA was isolated with miRNAeasy kit (Qiagen) and treated with TurboDNAse (Ambion) for 40 min at 37 °C in a final volume of 20 μL. DNAse was inactivated by incubating samples with DNAse inactivating reagent (Ambion) for 20 min on a shaker at 37 °C and diluted 5 times in water. Next, 7.7 μL of the diluted DNAse-treated RNA was used for the cDNA synthesis and subsequent qPCR. Reverse transcriptase reactions contained 7.7 μL of purified DNAse-treated total RNA, 2.5 μM random hexamer primers, 1 × RT buffer, 0.25 mM each of dNTPs, 3.33 U/μl MultiScribe reverse transcriptase and 0.25 U/μl RNase inhibitor. RT reaction (final volume of 20 μL) were incubated for 10 min at 25 °C, 30 min at 48 °C, 5 min at 85 °C and then held at 4 °C. RT products were was further diluted ten times with RNAse-free water. All TaqMan probes and primers were designed using online Primer3 software and are listed in Supplementary Material P1. qPCR primers and TaqMan probe for the Neomycin gene (NeoR) have been described before^36^. The levels of human AGO1, AGO2, AGO3 and AGO4 mRNAs were measured using individual TaqMan Gene Expression Assays (Applied Biosystems) - Hs01084653_m1 for AGO2, Hs00293044_m1 for AGO2, Hs00227461_m1 for AGO3 and Hs01059731_m1 for AGO4 taking into account the differences in qPCR primers efficiencies. Real-time PCR was performed in a 10 μL PCR reaction and included 2 μl of diluted RT product, 1x TaqMan Universal PCR Master Mix (Applied Biosystems), 900 nM of each primer and 250 nM TaqMan probe. The reactions were incubated in 384-well plates at 95 °C for 10 min, followed by 50 cycles of 95 °C for 15 sec and 60 °C for 1 min. All reactions were run in duplicate (or triplicate). Real-time PCR was performed using the LightCycler 480 Real-Time PCR System (Roche). Data was analysed with the LightCycler 480 software (Roche), determining the threshold cycle (Ct) by the second derivative max method. The efficiencies of all TaqMan Assays were assumed to be equal 2, and data was analysed using ΔΔCt method after normalization to NeoR gene (for overexpressed mRNAs).

### Western blot analysis

Cultured U2OS cells transfected with corresponding plasmid DNA (or U2OS cytoplasmic or nuclear lysates) were lysed in Laemmli loading buffer (250 mM Tris–HCl pH 6.4; 2% SDS; 100 nM b-MeEtOH), separated SDS-PAGE and transferred to nitrocellulose membranes. Membranes were blocked in Odyssey blocking buffer (LI-COR) or 5% non-fat milk containing 1xPBST for 1 h at RT. Incubation with anti-FLAG (Rockland, cat.200-301-383 S), anti-LaminB1 + B2 (Abcam, clone X223), anti-AGO1 (Sigma-Aldrich, clone 4B8), anti-EGFP (Santa Cruz, clone B-2), anti-α-tubulin (Sigma-Aldrich, clone AA13), anti-β-actin (Santa Cruz, clone C-4), anti-GAPDH (BioLegend, clone FF26A/F9) was performed at 4 °C overnight in a 1:1000 dilution. After incubation with primary antibodies, membranes were washed twice in 1 × PBST for 10 min and incubated for 1 h at RT with infrared fluorescent anti-mouse IgG (Pierce, cat.SA5-10176) or anti-rat IgG (Pierce, cat.SA5-10024) diluted 1:10000 in Odyssey blocking buffer (LI-COR); or HRP-conjugated anti-mouse IgG (Cell Signaling, cat. 7076) diluted 1:1000 in 5% non-fat milk containing 1xPBST. Membranes were washed four times for 10 min in PBST and signals were then acquired with Odyssey infrared imaging system (LI-COR) or luminescent detection.

## Additional Information

**How to cite this article**: Turchinovich, A. *et al*. Interference in transcription of overexpressed genes by promoter-proximal downstream sequences. *Sci. Rep*. **6**, 30735; doi: 10.1038/srep30735 (2016).

## Supplementary Material

Supplementary Information
