# Supplementary material for: Interference in transcription of overexpressed genes by promoter-proximal downstream sequences
Source: Sci Rep. 2016 Aug 3;6:30735. doi: 10.1038/srep30735 (PMC4971500; doi:10.1038/srep30735)
Supplement: Supplementary Information [file srep30735-s1.pdf]

SUPPLEMENTARY MATERIAL

Interference in transcription of overexpressed genes by promoter-proximal downstream sequences

Turchinovich A [1,2], Surowy HM [1,2], Tonevitsky A[3] and Burwinkel B[1,2]*

** corresponding author*
Email: a.turchinovich@dkfz.de
Tel: +49 6221 421461
Fax: +49 6221 421464

1 - Molecular Epidemiology C080**,** German Cancer Research Center**,** Im Neuenheimer Feld 581**,** Heidelberg, Germany
2 - Molecular Biology of Breast Cancer**,** Department of Gynecology and Obstetrics**,** Im Neuenheimer Feld 440, Heidelberg, Germany
3 - Hertsen Federal Medical Research Centre of the Ministry of Health of the Russian Federation, Moscow, Russia

A

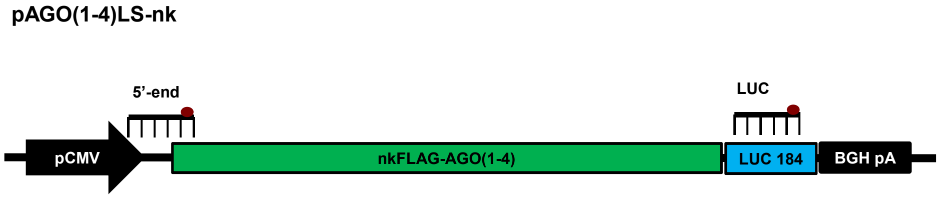

B

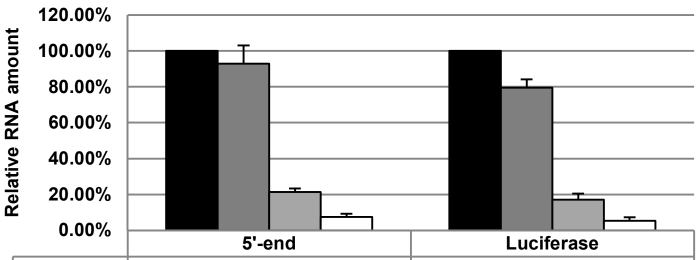

	5'-end	Luciferase
■pAGO1LS	100.00%	100.00%
■pAGO2LS	93.00%	79.58%
■pAGO3LS	21.43%	17.15%
□pAGO4LS	7.53%	5.34%

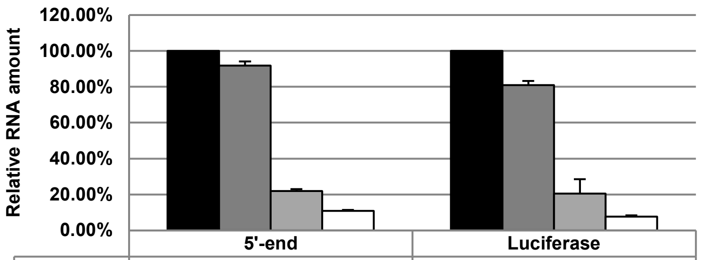

	5'-end	Luciferase
■pAGO1LS	100.00%	100.00%
■pAGO2LS	91.88%	80.97%
■pAGO3LS	21.94%	20.43%
□pAGO4LS	10.78%	7.60%

Supplementary Material 1. A. Structure of pAGO1LS-nk, pAGO2LS-nk, pAGO3LS-nk and pAGO4LS-nk plasmids encoding protein-null FLAG-AGO proteins, and the location of qPCR TaqMan Assays. **B.** TaqMan qPCR analysis of recombinant mRNAs generated from pAGO(1-4)LS-nk plasmids in MCF7 and HeLa cells 24 h after transfection. Data is presented as mRNA levels relative to pAGO1LS-nk and normalized on the NeoR mRNA levels for each construct. Each bar represents mean+S.D of three independent transfections.

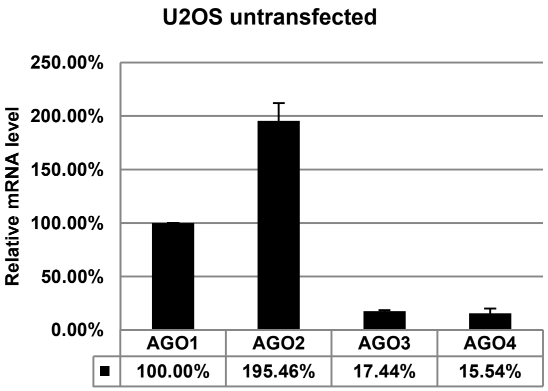

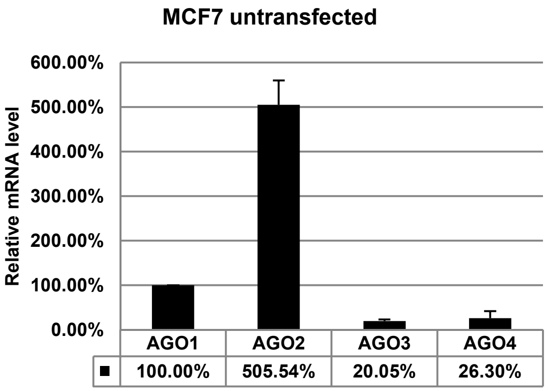

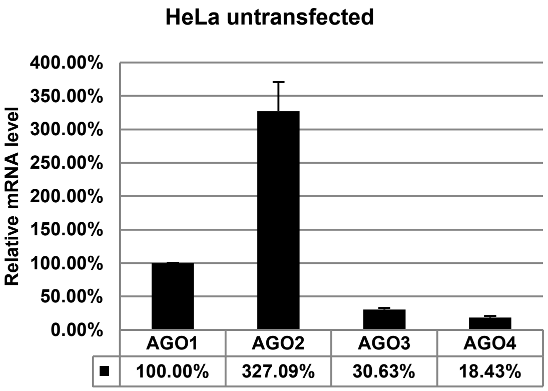

Supplementary Material 2. Relative expression of four human AGO1, AGO2, AGO3 and AGO4 mRNAs in U2OS, MCF7 and HeLa cell lines measured by AGO-specific TaqMan qPCR Assays (Applied Biosystems: Hs01084653_m1 for AGO1, Hs00293044_m1 for AGO2, Hs00227461_m1 for AGO3 and Hs01059731_m1 for AGO4). qPCR efficiencies of all TaqMan assays was considered to be equal 2. Data is presented as a fold change compared to AGO1 mRNA, each bar represents mean+S.D of three independent RNA isolations

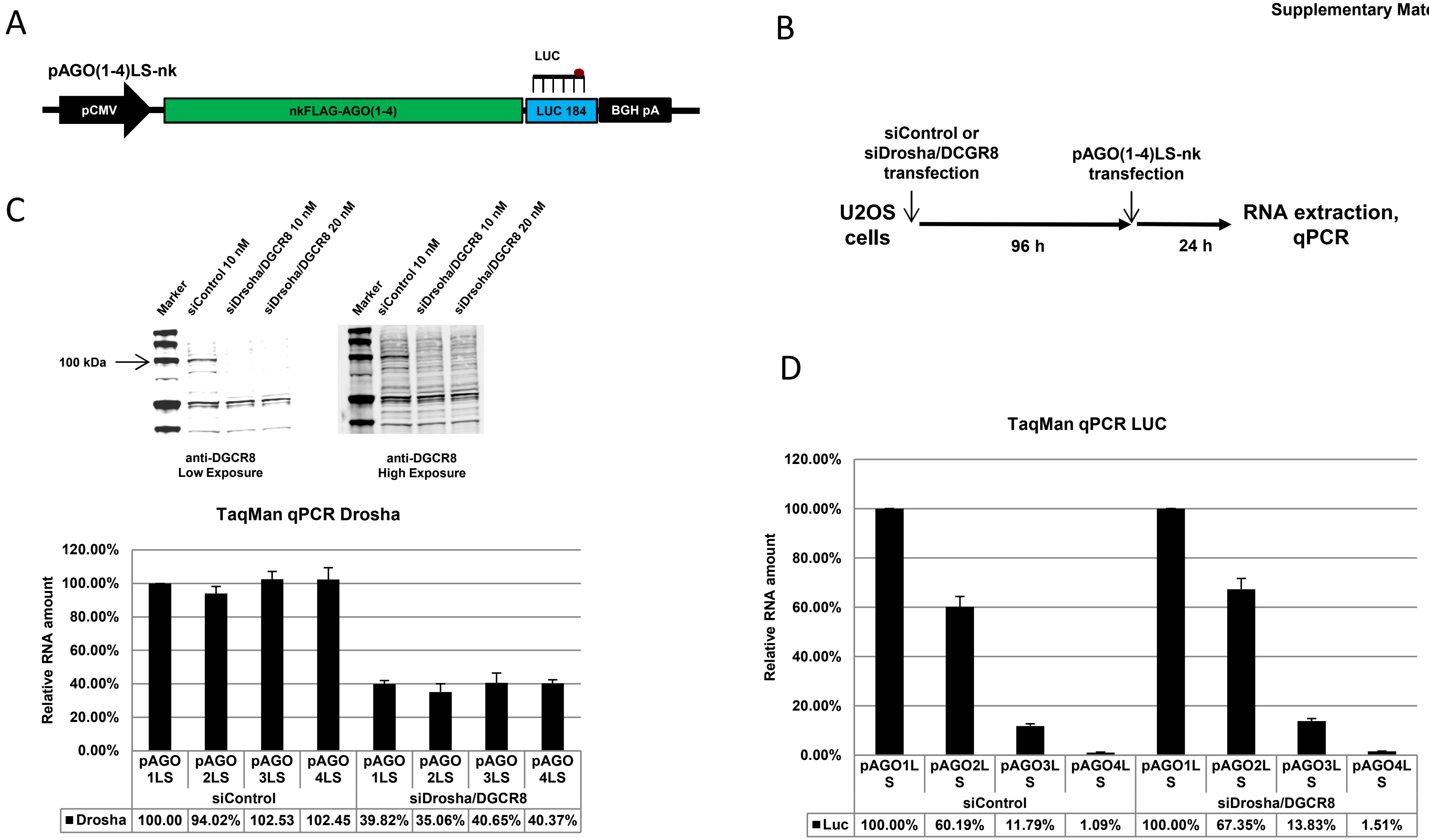

Supplementary Material 3. A. Structure of pAGO1LS-nk, pAGO2LS-nk, pAGO3LS-nk and pAGO4LS-nk plasmids encoding protein-null FLAG-AGO proteins, and the location of qPCR TaqMan Assays**. B.** Schematic representation of experimental workflow. U2OS cells were transfected with either siControl or equimolar mixture of siDrosha and siDGCR8 siRNA smart pools (Dharmacon) at final concentrations of 10 nM (replicate 1) or 20 nM (replicate 2) using siLentFect Lipid Reagent (Bio-Rad) 96h before transfection with pAGO1LS-nk, pAGO2LS-nk, pAGO3LS-nk and pAGO4LS-nk plasmids. The adherent cells were trypsinated 72h after transfection with siRNA, seeded on 24-well plates and let to recover for 24h before transfecting with plasmid DNAs. Finally, 24 h after transfection with plasmids, the total RNA and protein were collected. The relative expression of ectopic AGO(1-4) transcripts and endogenous Drosha mRNA were analyzed with corresponding TaqMan qPCR Assays. The knockdown of DGCR8 protein was analyzed only on western immunoblotting. **C.** *Upper part:* Western immunoblot performed on U2OS cells 96h after transfection with siControl (10 nM) or siDrosha/DGCR8 (10 nM or 20 nM) using an equimolar mixture of C-terminus and N-terminus specific anti-DGCR8 antibody (Sigma, cat. SAB4200088 and cat. SAB4200089). *Lower part:* TaqMan qPCR analysis of relative expression of Drosha mRNA after transfection of U2OS cells with pAGO(1-4)LS-nk and siDrosha/DGCR8. Data is presented as mRNA levels relative to Drosha mRNA amount in pAGO1LS+siControl transfected cells (taken as 100%) and normalized on Actin mRNA levels for each samples. Each bar represents mean+S.D of two replicates (10 nM or 20 nM siDrosha/DGCR8). **D.** TaqMan qPCR analysis of relative overexpression of recombinant Argonaute transcripts 24 h after transfection of U2OS cells with pAGO(1-4)LS-nk and either siControl or siDrosha/DGCR8. Each bar represents mean+S.D of two replicates. Note, simultaneous silencing of microprocessor components Drosha and DGCR8 did not affect the pattern of Argonautes overexpression in U2OS cells.

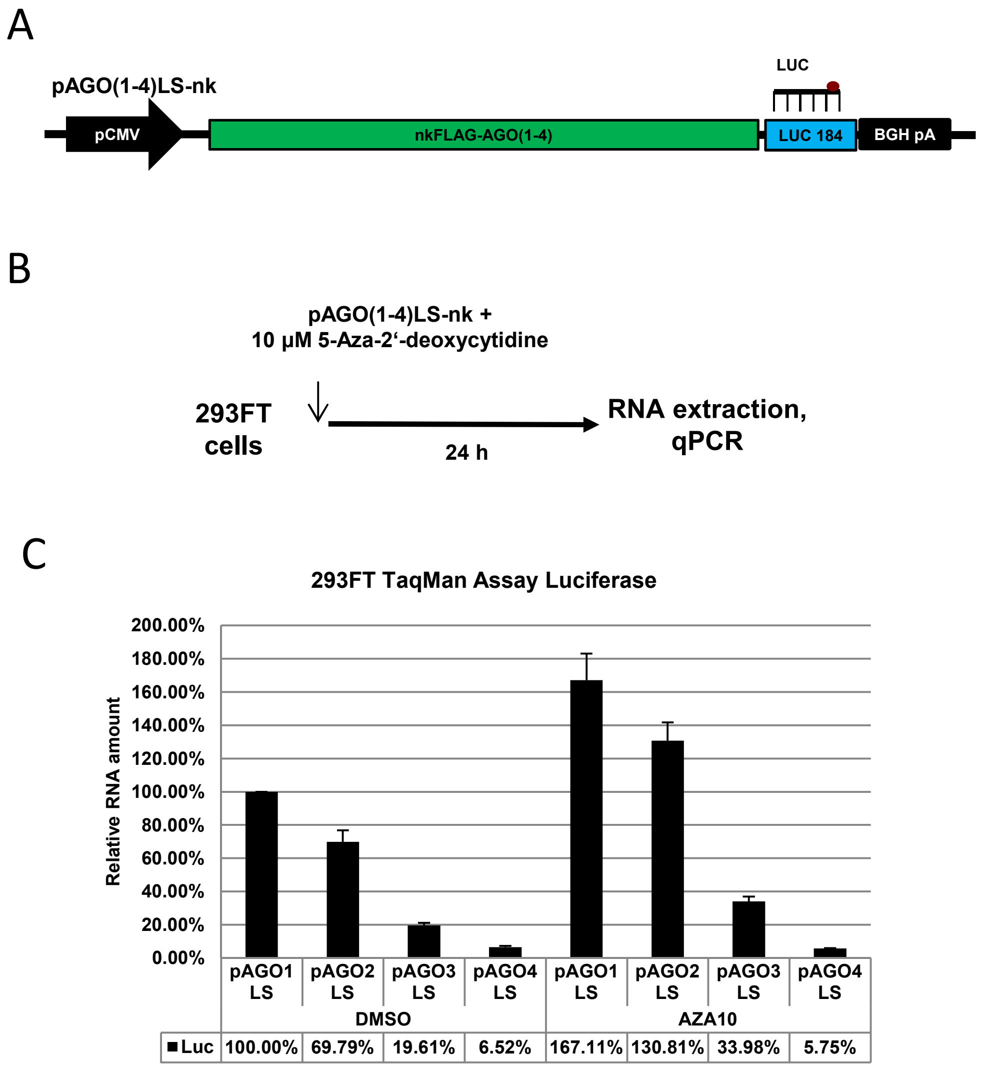

Supplementary Material 4. A. Structure of pAGO1LS-nk, pAGO2LS-nk, pAGO3LS-nk and pAGO4LS-nk plasmids encoding protein-null FLAG-AGO proteins, and the location of qPCR TaqMan Assays**. B.** Schematic representation of experimental workflow. 293FT cells were treated with transfection mix containing one of the pAGO(1-4)LS-nk plasmids and 10 µM 5-aza-2'-deoxycytidine for 24 h before RNA isolation and subsequent qRT-PCR analysis. **C.** TaqMan qPCR analysis of relative overexpression of exogenous AGO cDNAs 24 h after transfection of cells with pAGO(1-4)LS and either DMSO or 10 µM 5-aza-2'-deoxycytidine. Data is presented as mRNA levels relative to transcripts in pAGO1LS-nk+DMSO transfected cells (taken as 100%) and normalized on NeoR mRNA levels. Each bar represents mean+S.D of three independent transfections/treatments. Note, treatment with the demethylating agent 5-aza-2'-deoxycytidine (which supposed to block possible methylation of CMV promoter) did not significantly affect patterns of Argonautes overexpression in 293FT cells. Higher expression of AGO1, AGO2 and AGO3 transcripts in 10 µM 5-aza-2'-deoxycytidine treated 293FT cells compared to DMSO indicate that methylation of ectopic CMV promoter may prevent overexpression from this promoter to a minor extent in this cells.

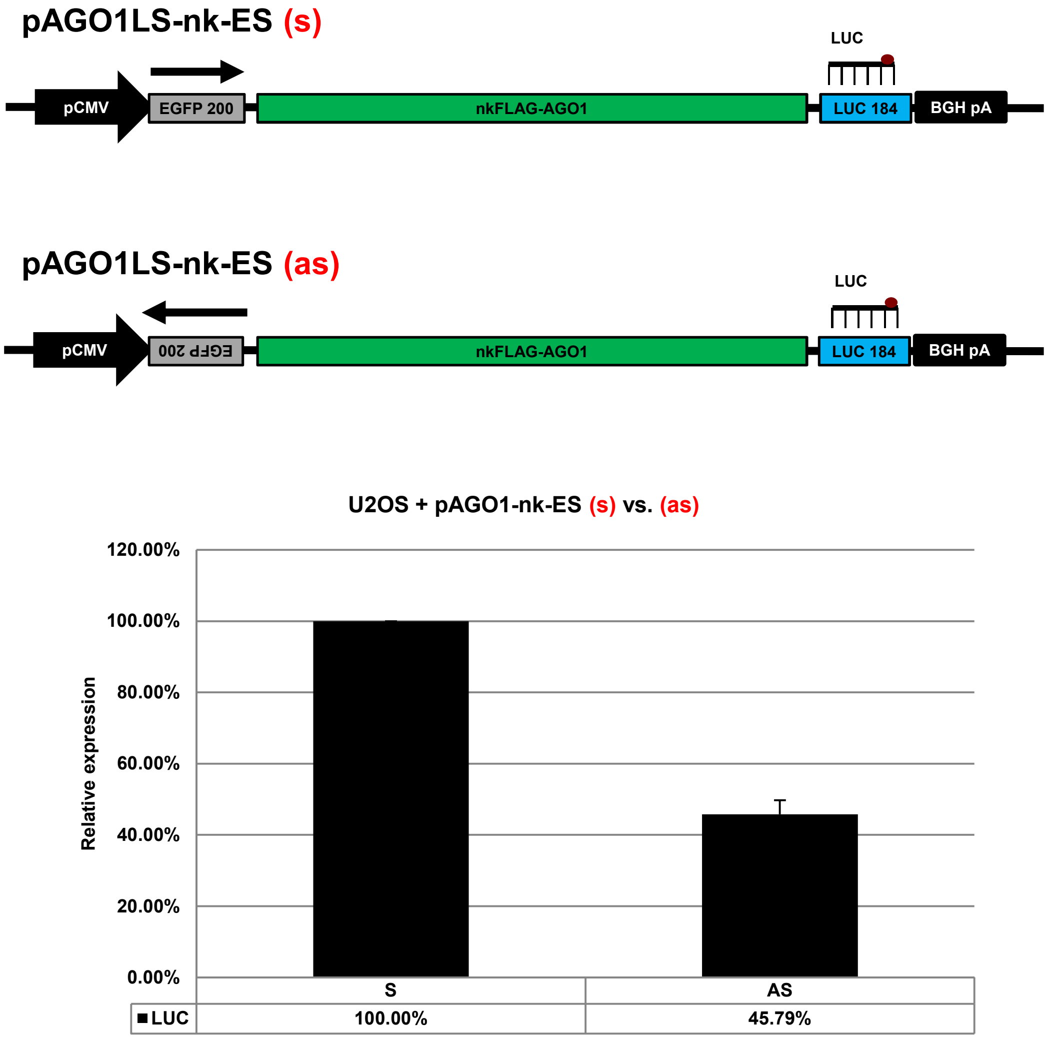

Supplementary Material 5. Structure of pAGO1LS-nk-ES containing short 200 bp fragment of EGFP gene right upstream the protein-null FLAG-AGO1 sequences either in sense (s) or antisense (as) orientation; and TaqMan qPCR analysis of recombinant mRNAs generated from those plasmids in U2OS cells 24 h after transfection. The location of the 3'-end (luciferase) TaqMan qPCR assays is indicated on the plasmid map. Data is presented as a graph combining relative overexpression efficacy of transcripts from plasmids with sense EGFP orientation (taken as 100%) vs. transcripts containing EGFP in antisense orientation. Each bar represents mean+S.D of three independent transfections. Note, significant dependence of transcription depending on the orientation of short 200 bp EGFP fragment directly downstream the promoter.

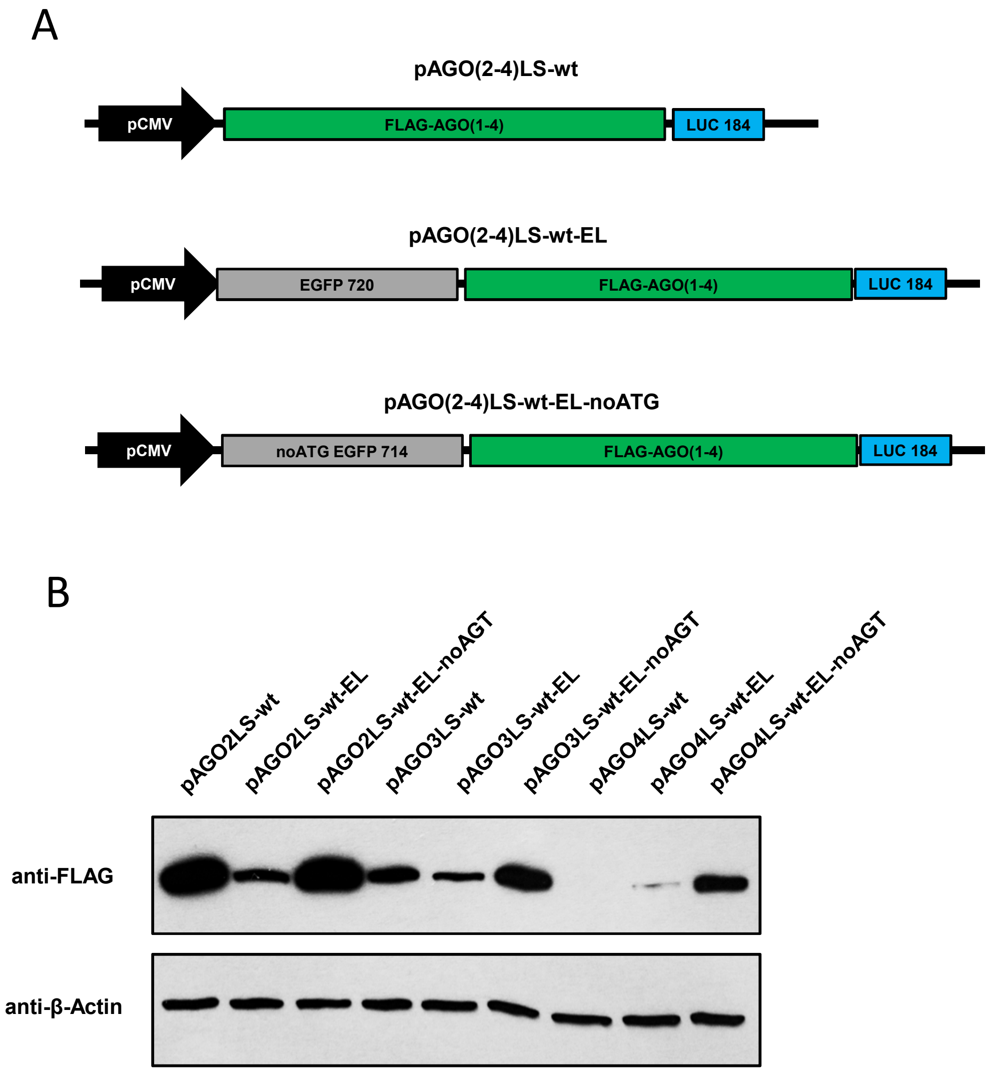

Supplementary Material 6. A. Structure of parental pAGO(2-4)LS-wt and their derivatives: pAGO(2-4)LS-wt-EL - carrying 720 bp protein-null EGFP coding sequence (w/o the first ATG codon and the middle ORF ATG codon) upstream the FLAG-AGOs coding sequences and pAGO(2-4)LS-wt-EL-noATG - carrying 714 bp EGFP coding sequence (w/o all ATG codons). **B.** Western immunoblot performed with anti-FLAG antibody showing relative FLAG-AGO2, FLAG-AGO2, FLAG-AGO3 and FLAG-AGO4 proteins expression in U2OS cells 24 h after transfection with pAGO(2-4)LS-wt, pAGO(2-4)LS-wt-EL and pAGO(2-4)LS-wt-ELnoATG constructs. Note, recombinant proteins production is significantly impaired when upstream EGFP fragments contained ATG codons.

A

AGO1 vs AGO2 = 75.5%	AGO1i vs AGO2i = 72.3%	AGO1ii vs AGO2ii = 75.8%	AGO1iii vs AGO2iii = 80.3%
AGO1 vs AGO3 = 73.0%	AGO1i vs AGO3i = 68.4%	AGO1ii vs AGO3ii = 76.4%	AGO1iii vs AGO3iii = 74.7%
AGO1 vs AGO4 = 73.5%	AGO1i vs AGO4i = 72.7%	AGO1ii vs AGO4ii = 75.3%	AGO1iii vs AGO4iii = 74.4%
AGO2 vs AGO3 = 70.7%	AGO2i vs AGO3i = 67.3%	AGO2ii vs AGO3ii = 72.9%	AGO2iii vs AGO3iii = 72.5%
AGO2 vs AGO4 = 70.2%	AGO2i vs AGO4i = 68.6%	AGO2ii vs AGO4ii = 72.3%	AGO2iii vs AGO4iii = 73.0%
AGO3 vs AGO4 = 73.8%	AGO3i vs AGO4i = 70.7%	AGO3ii vs AGO4ii = 77.1%	AGO3iii vs AGO4iii = 77.6%

B

```
            40        50        60        70        80        90
AGO3 ii  GCCACTAGAAGTCTGTAATATTGTGGCAGGGCAACGATGTATCAAGAAGCTAACAGACAA
         :::::: :: ::::::::::: :::::::: :: :: ::::::::::::::::: :::::::::
AGO4 ii  GCCACTCGAGGTCTGTAATATAGTGGCAGGACAGCGATGTATCAAGAAGCTCACAGACAA
                 10        20        30        40        50        60

           100       110       120       130       140       150
AGO3 ii  TCAGACTTCCACTATGATCAAGGCAACAGCAAGATCTGCACCAGATAGACAAGAGGAAAT
         :::::::::::: ::::::: :: :::::::::::::: :: :: ::::: :: :: ::
AGO4 ii  TCAGACTTCCACAATGATCAAAGCTACAGCAAGATCTGCTCCTGACAGACAGGAAGAGAT
                 70        80        90       100       110       120

           160       170        180              190       200
AGO3 ii  TAGCAGATTGGTAAGAAGTGCAA-ATTATGA-------AACAGATCCATTTGTTCAGGAG
          :: ::: ::::  :::: :::: : ::::        : : :::::::   :: : ::
AGO4 ii  CAGTAGACTGGT--GAAGAGCAACAGTATGGTGGGTGGACCTGATCCATACCTTAAAGAA
                130         140       150       160       170

         210       220       230       240       250       260
AGO3 ii  TTTCAATTTAAAGTTCGGGATGAAATGGCTCATGTAACTGGACGCGTACTTCCAGCACCT
         :::    ::   :: :   :::::::: :  :  : :: ::  : :::::::::::::: 
AGO4 ii  TTTGGTATTGTTGTCCACAATGAAATGACAGAGCTCACAGGCAGGGTACTTCCAGCACCA
        180       190       200       210       220       230

         270       280       290       300       310       320
AGO3 ii  ATGCTCCAGTATGGGGGACGGAATCGGACAGTAGCAACACCGAGCCATGGAGTATGGGAC
         ::::: :: ::::: :: ::::::   :::::::: ::::: : ::: :: :: ::::::
AGO4 ii  ATGCTGCAATATGGAGGCCGGAATAAAACAGTAGCCACACCCAACCAGGGTGTCTGGGAC
        240       250       260       270       280       290

         330       340       350       360       370       380
AGO3 ii  ATGCGAGGGAAACAATTCCACACAGGAGTTGAAATCAAAATGTGGGCTATCGCTTGTTTT
         :::::::: :: :: ::  :  : ::  ::::::: ::: : :::::  : :::::::::
AGO4 ii  ATGCGAGGAAAGCAGTTTTATGCTGGCATTGAAATTAAAGTTTGGGCAGTTGCTTGTTTT
        300       310       320       330       340       350

         390       400       410       420       430       440
AGO3 ii  GCCACACAGAGGCAGTGCAGAGAAGAAATATTGAAGGGTTTCACAGACCAGCTGCGTAAG
         ::  : ::::  :: :: :: :::::  :: : ::: :::::::: :::::::::::::: 
AGO4 ii  GCACCTCAGAAACAATGTAGGGAAGATTTACTAAAGAGTTTCACTGACCAGCTGCGTAAA
        360       370       380       390       400       410

         450       460       470       480       490       500
AGO3 ii  ATTTCTAAGGATGCAGGGATGCCCATCCAGGGCCAGCCATGCTTCTGCAAATATGCACAG
         :: :::::::::::::: :::::::::::::: ::::::::: :::::::: :::::::: 
AGO4 ii  ATCTCTAAGGATGCAGGAATGCCCATCCAGGGTCAGCCATGTTTCTGCAAGTATGCACAA
        420       430       440       450       460       470

         510       520       530       540       550       560
AGO3 ii  GGGGCAGACAGCGTAGAGCCCATGTTCCGGCATCTCAAGAACACATATTCTGGCCTACAG
         :: :::::::: :: ::::: :::::    ::::: :: :  :: :::   :::::::::
AGO4 ii  GGTGCAGACAGTGTGGAGCCTATGTTTAAACATCTGAAAATGACTTATGTGGGCCTACAG
        480       490       500       510       520       530

         570       580       590       600       610       620
AGO3 ii  CTTATTATCGTCATCCTGCCGGGGAAGACACCAGTGTATGCGGAAGTGAAACGTGTAGGA
         :: ::  : :: :::::::: :: ::::::::::: ::::::::: :::::::::::: :::
AGO4 ii  CTAATAGTGGTTATCCTGCCTGGAAAGACACCAGTATATGCGGAGGTGAAACGTGTTGGA
        540       550       560       570       580       590

         630       640       650       660       670       680
AGO3 ii  GACACACTTTTGGGTATGGCTACACAATGTGTTCAAGTCAAGAATGTAATAAAAACATCT
         :: :: ::: : :::::::: ::::: ::::: :: :: :: :::::: : :: :: :: 
AGO4 ii  GATACCCTTCTAGGTATGGCCACACAGTGTGTCCAGGTAAAAAATGTAGTGAAGACCTCA
        600       610       620       630       640       650

         690       700       710       720       730       740
AGO3 ii  CCTCAAACTCTGTCAAACTTGTGCCTAAAGATAAATGTTAAACTCGGAGGGATCAATAAT
         :::::::: :: :: ::  : ::::: :::::::::   ::::: ::::: :: :: :::
AGO4 ii  CCTCAAACCCTTTCCAATCTTTGCCTGAAGATAAATGCAAAACTTGGAGGAATTAACAAT
        660       670       680       690       700       710

         750       760       770       780       790       800
AGO3 ii  ATTCTTGTACCTCATCAAAGACCTTCTGTGTTCCAGCAACCAGTGATCTTTTTGGGAGCC
          : ::::: :::::::::::: :: :: :::::::::::: :: :: :::::  ::::::: 
AGO4 ii  GTGCTTGTGCCTCATCAAAGGCCCTCGGTGTTCCAGCAGCCTGTCATCTTCCTGGGAGCG
        720       730       740       750       760       770

         810       820       830       840       850       860
AGO3 ii  GATGTCACTCATCCACCTGCTGGTGATGGAAAGAAGCCTTCTATTGCTGCTGTTGTAGGT
         :::::::: :: :: :: :: :: ::::: ::::: ::::: ::::::::::: :: :: 
AGO4 ii  GATGTCACACACCCCCCAGCAGGGGATGGGAAGAAACCTTCCATTGCTGCTGTGGTTGGC
        780       790       800       810       820       830

         870       880       890       900       910       920
AGO3 ii  AGTATGGATGCACACCCAAGCAGATACTGTGCCACAGTAAGAGTTCAGAGACCCCGACAG
         ::::::::::  ::::: ::: : ::::::::::: ::  : :: ::::   :::: :::
AGO4 ii  AGTATGGATGGCCACCCCAGCCGGTACTGTGCCACCGTTCGGGTGCAGACTTCCCGGCAG
        840       850       860       870       880       890

         930       940       950
AGO3 ii  GAGATCATCCAGGACTTGGCCTCCATGGTC
         ::::::  ::: ::  :  :::: :  :::
AGO4 ii  GAGATCTCCCAAGAGCT--CCTCTACAGTC
        900       910         920
```

Supplementary Material 7. A. Percentage of sequence identity calculated after alignment of corresponding Argonaute sequences. **B.** Alignment of AGO3ii and AGO4ii regions.

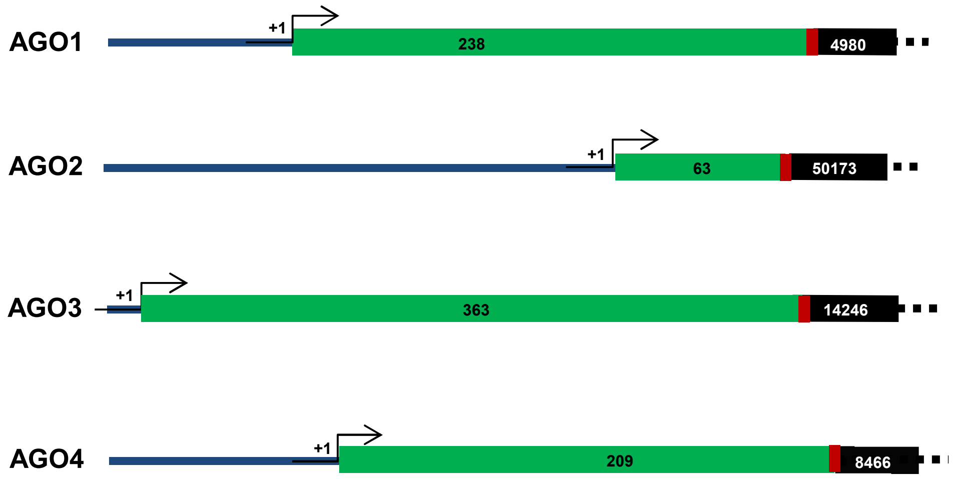

Supplementary Material 8. The location and the length of first exons (green boxes) and the first introns (black boxes) of human AGO1, AGO2, AGO3 and AGO4 genes relative to their TSSs. The length is indicates in base pairs within each box. The location of the 19 bp part of Argonautes coding sequences are indicated in red box. Endogenous human AGO sequences are located in 19 separate exons; and only 19 nucleotides of their coding sequences are located in the first exon in a relatively close proximity from the transcription start site (TSS) (344 bp for AGO3 and 190 bp for AGO4). Second exons, carrying another 182 bp (for AGO3) and 173 nt (for AGO4) of the coding sequences are located 14246 nt and 8466 nt downstream.

Supplementary Material P1

Primers and Probes

FLAG-AGO cloning (XbaI/EcoRI)

**pAGO(1-4)LS-wt**	
AGO forward xbaI	gatatctaga cgatatcgccgccgccatg
AGO reverse	ctggcggccgttactagtggatccactgaattc

No Kozak FLAG-AGO cloning (XbaI/EcoRI)

**pAGO(1-4)LS-nk**	
AGO NK forward xbaI	gata tctaga ggactacaaggacgacgatg
AGO reverse	ctggcggccgttactagtggatccactgaattc

Luciferase Fragments cloning (EcoRI/BamHI)

**pAGO(1-4)LS**	
Lucs Forward EcoRI	ggcc gaa ttc tga acg tgc aaa
Lucs Reverse BamHI	gaga gga tcc gga gtt cat gat
**pAGO(1-4)LL**	
LucL Forward EcoRI	ggcc gaa ttc atg gaa gac gcc aaa aac
LucL Reverse BamHI	gaga gga tcc tta cac ggc gat ctt tcc

Luciferase Fragments cloning (XbaI)

**pAGO(1-4)LUL**	
Luc Forward XbaI	gata tctaga gaa gac gcc aaa aac ata
Luc Reverse XbaI	gata tctaga tta cac ggc gat ctt tcc

Deletion mutagenesis primers

**pdelAGO1(i-iii)**	
Ago1for1	phos-cag gaa caa aag cat acc tac
Ago1rev1	phos-cat gcg gcc gct agc gta atc
Ago1for2	phos-tac aag tcc acc cgt ttc aag
Ago1rev2	phos-gcc aac ttg tag gca ggg cag
Ago1for3	phos-gcc gtg cag gtt cac cag gat
Ago1rev3	phos-gaa ttg gat gag gag ctc acg
**pdelAGO2(i-iii)**	
Ago2for1	phos-agg cac aag ttg gtt ctg cgc
Ago2rev1	phos-cat gcg gcc gct agc gta atc
Ago2for2	phos-gtg cag cag cac cgg cag gag
Ago2rev2	phos-gtc ctt gaa ata ctg ggc cac
Ago2for3	phos-gcc aag gcg gtc cag gtt cac
Ago2rev3	phos-gcg cac ggt ggc gca gta gcg
**pdelAGO3(i-iii)**	
Ago3for1	phos-caa gtc ggg cag gaa cag aaa cac
Ago3rev1	phos-cat gcg gcc gct agc gta atc
Ago3for2	phos-att caa ttt tat aag tca act cgg
Ago3rev2	phos-cag aca ggg aag gtg cgg gta ctt
Ago3for3	phos-ctt gcc aag gct gta cag att cac
Ago3rev3	phos-aag aag ttc ccg gac cat gga ggc
**pdelAGO4(i-iii)**	
Ago4for1	phos-cca ctc gag gtc tgt aat ata gtg
Ago4rev1	phos-cat gcg gcc gct agc gta atc
Ago4for2	phos-ctg att cag ttc tac aaa tcc aca
Ago4rev2	phos-caa gta tgt atg ctt ttg ttc ttg
Ago4for3	phos-gcc ttg gct aag gct gtg caa atc
Ago4rev3	phos-cag ctc tcg aac cat gtt agt cag

AGO Fragments cloning (XbaI/EcoRI)

pAGO1i, pAGO1ii, pAGO1iii

AGO NK forward xbaI	gata tctaga ggactacaaggacgacgatg
AGO1iR	ggcc gaattc gccaacttgtaggcagggcag
AGO1iiF	gata tctaga cag gaa caa aag cat acc tac
AGO1iiR	ggcc gaattc gaa ttg gat gag gag ctc acg
AGO1iiiF	gata tctaga tacaagtccacccgtttcaag
AGO reverse	ctggcggccgttactagtggatccactgaattc
**pAGO2i, pAGO2ii, pAGO2iii**	
AGO NK forward xbaI	gata tctaga ggactacaaggacgacgatg
AGO2iR	ggcc gaattc gtc ctt gaa ata ctg ggc cac
AGO2iiF	gata tctaga agg cac aag ttg gtt ctg cgc tac
AGO2iiR	ggcc gaattc gcg cac ggt ggc gca gta gcg
AGO2iiiF	gata tctaga gtg cag cag cac cgg cag gag
AGO reverse	ctggcggccgttactagtggatccactgaattc
**pAGO3i, pAGO3ii, pAGO3iii**	
AGO NK forward xbaI	gata tctaga ggactacaaggacgacgatg
AGO3iR	ggcc gaattc cagacagggaaggtgcgggt
AGO3iiF	gata tctaga caagtcgggcaggaacagaaac
AGO3iiR	ggcc gaattc aagaagttcccggaccatggag
AGO3iiiF	gata tctaga ttcaattttataagtcaactc
AGO reverse	ctggcggccgttactagtggatccactgaattc
**pAGO4i, pAGO4ii, pAGO4iii**	
AGO NK forward xbaI	gata tctaga ggactacaaggacgacgatg
AGO4iR	ggcc gaattc caagtatgtatgcttttgttc
AGO4iiF	gata tctaga ccactcgaggtctgtaatatag
AGO4iiR	ggcc gaattc cagctctcgaaccatgttagtc
AGO4iiiF	gata tctaga ctgattcagttctacaaatcc
AGO reverse	ctggcggccgttactagtggatccactgaattc

EFGP Fragments cloning (XbaI)

**pAGO(1-4)LS-nk-ES**	
EGFPs Forward XbaI	gata tctaga atg ccc gaa ggc tac gtc
EGFPs Reverse XbaI	gata tctaga gcc atg ata tag acg ttg
**pAGO(1-4)LS-nk-EL**	
EGFPL Forward XbaI	gata tctaga gtg agc aag ggc gag gag
EGFPL Reverse XbaI	gata tctaga tta ctt gta cag ctc gtc

EFGP mutagenesis primers

KillORFf:	phos-tgc ccg aag gct acg tcc agg
KillORFr:	phos-ggc gga ctt gaa gaa gtc gtg

UBC promoter cloning

UBCmluF:	gaga acgcgt gtg tcg gct cca gat ctg
UBCxbaRS:	ggcc tctaga gtg acg atc aca gcg atc
UBCxbaRL:	ggcc tctaga gtc taa caa aaa agc caa aaa cg

E1α promoter cloning

EPmluF:	gaga acgcgt cgt gag gct ccg gtg ccc gt
EPxbaRS:	ggcc tctaga tgt gtt ctg gcg gca aac ccg
EPxbaRLc:	ggcc tctaga ctc acg aca cct gaa atg gaa

mPGK promoter cloning

PGKmluF:	gaga acgcgt atg gtc gag tac cgg gta g
PGKxbaRL:	ggcc tctaga ccg cta gag gtc gaa agg

TK promoter cloning

TKfmlu:	gaga acgcgt aaa tga gtc ttc gga cct
TKrxba:	ggcc tctaga ccc agt gcc tca cga cca

CMV enhancer mutagenesis primers

KillCMVeF:	phos-gtg atg cgg ttt tgg cag tac
KillCMVeR:	phos-cgg aac tcc ata tat ggg cta

5'-end TaqMan Assay

5'-end forward	CCAAGCTGGCTAGCGTTTA
5'-end reverse	TAATCGGGCACGTCATAAGG
5'-end probe	FAM-CGGGCCCTCTAGAGGACTACAAGGA-TAMRA

EGFP TaqMan Assay

EGFP forward	ACGACGGCAACTACAAGACC
EGFP reverse	GTCCTCCTTGAAGTCGATGC
EGFP probe	FAM-CGACACCCTGGTGAACCGCA-TAMRA

Luciferase TaqMan Assay

Luciferase forward	CCAGGGATTTCAGTCGATGT
Luciferase reverse	GGACTCTGGCACAAAATCGT
Luciferase probe	FAM-TCGTCACATCTCATCTACCTCCCGG-TAMRA

NeoR TaqMan Assay

NeoR forward	CTCCTGCCGAGAAAGTATCCA
NeoR reverse	GCCGGATCAAGCGTATGC
NeoR probe	FAM-CGCCGCATTGCATCAGCCAT-TAMRA
